# Comparative analysis of RAD-seq methods for SNP discovery and genetic diversity assessment in oil seed crop safflower

**DOI:** 10.1038/s41598-025-06706-2

**Published:** 2025-07-02

**Authors:** Pooja Pathania, Gaddam Prasanna Kumar, Nishu Gupta, R. Parimalan, J. Radhamani, Rajesh Kumar, Sunil Shriram Gomashe, Palchamy Kadirvel, S. Rajkumar

**Affiliations:** 1 ICAR- National Bureau of Plant Genetic Resources, Pusa Campus, New Delhi, 110012 India; 2 ICAR- National Bureau of Plant Genetic Resources, RS-Akola, Akola, India; 3 ICAR-Indian Institute of Oilseeds Research, Rajendranagar, 500030 Hyderabad India

**Keywords:** *Carthamus tinctorius*, Genetic diversity, Genome sampling, RAD-seq, Restriction enzymes, SNP discovery, Molecular biology, Plant sciences

## Abstract

Safflower (*Carthamus tinctorius* L.) is an important oilseed crop with diverse uses and the potential for genetic improvement. This study aimed to optimize genotyping-by-sequencing (GBS) for safflower via in silico and in vitro methods with two restriction site-associated DNA sequencing (RAD-seq) approaches, i.e., single restriction site-associated DNA sequencing (sdRAD-seq) and double-digest RAD sequencing (ddRAD-seq) and three restriction enzyme combinations (ApeKI, NlaIII_Msel, and EcoRI_Msel). Forty-two safflower accessions were selected for this study. In silico testing revealed that NlaIII_Msel generated the largest number of DNA fragments, followed by ApeKI and EcoRI_Msel. The in vitro results showed that ddRAD-seq outperformed sdRAD-seq in terms of raw read count, alignment rate, depth and breadth of coverage, and SNP detection. An alignment-free analysis using k-mer counting and sketching based on genetic distance further confirmed the superiority of ddRAD-seq. Gene-level k-mer validation identified more core genes in the ddRAD-seq data. Variant calling resulted in 6,721, 173,212, and 221,805 single nucleotide polymorphic sites (SNPs) for ApeKI, NlaIII_Msel, and EcoRI_Msel, respectively. SNP annotation and distribution analysis revealed that EcoRI_Msel captured more SNPs with fewer missing observations. Principal component analysis via ddRAD-seq data explained 30.29% and 33.98% of the total genetic variation in NlaIII_Msel and EcoRI_Msel, respectively. This study demonstrated that ddRAD-seq with the EcoRI_Msel enzyme combination is the most suitable GBS approach for genome sampling and SNP genotyping in safflower.

**Supplementary Information:**

The online version contains supplementary material available at 10.1038/s41598-025-06706-2.

## Introduction

Safflower (*Carthamus tinctorius* L.), an oilseed crop from the family Asteraceae, is considered a versatile crop owing to its benefits and uses, and has significant potential for future exploitation in terms of its genetic diversity. Traditionally, safflower has been cultivated for its floral pigments (red and yellow), which are utilized in industries such as textiles, food, cosmetics, painting, and pharmaceuticals^1,2^. Safflower is also used as livestock feed^2^, seed oil for biodiesel production^3^, straw for biogas and bioethanol refineries^4,5^, and phytoremediation^1^. The commercial importance of safflower is related primarily to its fatty acid profile, which includes two unsaturated fatty acids (oleic acid and linoleic acid), accounting for approximately 90% of the total fatty acids, with the remaining 10% comprising saturated fatty acids such as palmitic acid and stearic acid^6^. Safflower is cultivated in more than 60 countries under diverse climatic conditions, with Kazakhstan, the USA, Mexico, India, Turkey, and China being the highest producers^7^. Safflower crops are also recognized for their tolerance to drought, high temperatures^8^, and salinity^9^.

DNA polymorphism resulting from restriction length fragment polymorphism (RLFP), simple sequence repeat (SSR), and single nucleotide polymorphism (SNP) forms the basis of modern genetics. The discovery and analysis of these genomic variations, such as SNPs, insertions/deletions (InDels), and structural variations (SVs), among individuals and between populations, constitute the foundation for recognizing different genotypes and linking them to phenotypes^10,11^. Reduced representation sequencing (RRS) is one of the most cost-effective and efficient high-throughput genotyping methods^12^. Current RRS methods include single restriction site-associated DNA sequencing (sdRAD-seq)^13^, double-digest RAD sequencing (ddRAD-seq)^14^ and Diversity Array Technology (DArT)^15^. Both methods reduce genome complexity via restriction enzymes (REs). The utilization of different single and paired restriction enzymes in various combinations renders these approaches more suitable for addressing diverse types of differences in the genomes of different crops. The selection of restriction enzymes is also crucial because it can affect genome coverage^12^ and SNP genotyping^16^. Compared with previous fragment size analysis methods or sequence comparisons of individual nuclear or chloroplast DNA regions, the genotyping-by-sequencing (GBS) approach has the potential to provide more precise relationships both within and among closely related species^17^.

The process of building GBS libraries involves restriction enzymes (REs) to reduce the complexity of the genome^18^, as well as a barcoding system to multiplex template DNA to produce representative libraries^13^. This genomic reduction, achieved via REs, represents a significant technical challenge because the genomic distribution of SNPs depends on the precise combination of the REs employed^19^. Selecting appropriate REs is crucial for species-specific GBS optimization. By utilizing suitable REs, repetitive regions of the genome can be avoided, and regions with fewer copies can be targeted with two to three times greater efficiency for accessing important regions of the genome^20^. When selecting optimal REs for GBS optimization, factors such as genome size, the methylation of repeat elements, and genome organization are considered^21^. In plants, the REs ApeKI, PstI, and EcoRI are generally utilized and combined with frequent cutter enzymes such as MspI, MseI, and HpaII, depending on the genomic specificity of each species^22^.

While various marker systems have proven effective in assessing genetic diversity in safflower, there is insufficient information to directly compare SNP markers to other systems for this crop. However, given the success of SNP markers in other species and their generally high informativeness, it is likely that they would also be valuable for safflower genetic diversity studies. SNP markers have shown substantial genetic variation and have been useful in identifying population structure and genetic diversity^23^. In the present study, two RAD-seq approaches (sdRAD-seq and ddRAD-seq) with three restriction enzyme combinations (ApeKI, NlaIII_Msel and EcoRI_Msel) were evaluated for GBS optimization via in silico and in vitro methods. The data were analysed utilizing both alignment-free and alignment-based approaches. The comparison between single-digest RAD-seq (sdRAD-seq) and double-digest RAD-seq (ddRAD-seq) was chosen to evaluate their relative performance and efficiency in genomic analysis. These methods were selected because they are widely used reduced representation sequencing techniques that allow for cost-effective genome-wide genotyping^24^. The main difference between these approaches lies in the number of restriction enzymes used: sdRAD-seq uses a single enzyme, while ddRAD-seq employs two enzymes. This distinction can significantly impact the resulting genomic coverage, number of loci sampled, and overall sequencing efficiency^25^. By comparing these methods, researchers can assess their relative strengths and limitations in various applications, such as SNP discovery, genetic mapping, and population genomics studies. Interestingly, while both methods have their merits, ddRAD-seq has gained popularity due to its flexibility and potential for more balanced genomic sampling^24,25^. However, the choice between sdRAD-seq and ddRAD-seq often depends on specific research goals, target species, and available resources. By directly comparing these methods, researchers can make informed decisions about which approach is most suitable for their particular study, considering factors such as genome size, desired marker density, and budget constraints.

## Materials and methods

### Materials

A total of 42 diverse safflower accessions were selected for the study (Supplementary Table S1) and were obtained from the National Gene Bank of the Indian Council of Agriculture Research, National Bureau of Plant Genetic Resources, New Delhi (ICAR-NBPGR). Of these, 19 accessions originated from different countries, whereas the remaining 23 accessions originated from various states of India. All the accessions were purified by selfing (single-seed descent method) for two generations, at the Akola, Regional Station of the NBPGR. Seeds were soaked overnight in water and germinated on towel paper for 5–7 days. The seedlings were subsequently subjected to DNA extraction using the DNeasy Plant Kit (Qiagen, USA). The quality and quantity of the extracted DNA were assessed by electrophoresis.

## Selection of restriction enzymes (REs)

Three pairs of enzymes were selected to evaluate the efficacy of the sdRADseq and ddRADseq methods. For sdRADseq, the ApeKI restriction enzyme was utilized, whereas, for ddRADseq, a combination of a rare and frequent cutter, specifically the EcoRI_Msel and NlaIII_Msel pair of restriction enzymes, was employed (Table 1). In vivo studies on number of restriction site mapping in the reference genome provided EcoRI and NlaIII were having higher number of sites along with Mse1 and also commonly used enzymes in RAD-seq due to their distinct recognition sites, whereas Msel was paired with later two enzymes for its compatibility^22^.

**Table 1:** Summary of the different restriction enzymes used in this study.

Enzyme	Recognition site	Length	Methylation sensitivity
ApeKI	G|CWGC	5	CpG
EcoRI	G|AATTC	6	CpG
NlaIII	CATG|	4	No
Msel	T|TAA	4	No

## In Silico simulation

In silico analyses of sdRADseq and ddRADseq methodologies using ApeKI, EcoRI_Msel, and NlaIII_Msel restriction enzymes were conducted via the R package simRAD^26^. These analyses were performed using safflower reference genome version 1 obtained from The Genome database of *Carthamus tinctorius*^27^.

## In vitro testing (sdRADseq and DdRADseq protocol)

In vitro testing was conducted on 42 diverse accessions common to both methods (sdRADseq and ddRADseq) via restriction enzymes (ApeKI, NlaIII_Msel, and EcoRI_Msel). The sdRAD-seq and ddRADseq protocols were similar, differing only in the digestion step with respect to the restriction enzymes used in this study. DNA samples (200 ng of DNA/sample) were digested with ApeKI for sdRAD-seq and double digested using rare-cutting NlaIII/EcoRI and frequent-cutting MseI enzymes (New England BioLabs, US) for ddRADseq. The digested DNA fragments were subsequently ligated with P1 and P2 adapters via T4 ligase (New England BioLabs, US). The ligation reaction involved overnight incubation (> 12 h) at room temperature (approximately 21 °C) followed by heat deactivation of the enzyme at 65 °C for 10 min. To eliminate unincorporated adapters and small DNA fragments (< 300 bp), the ligation products were purified using 0.8X volume of Agencourt AMPure XP SPRI magnetic beads (Beckman Coulter, US). A unique combination of dual-indexed barcodes was attached to the purified fragments through 14 PCR cycles. Indexed PCR products were pooled in equal volumes, and fragments between 300 and 700 bp in size were selected using Agencourt AMPure XP SPRI magnetic beads. The library concentration was determined using a Qubit.3 fluorometer (Life Technologies, US) with a Qubit dsDNA HS (High Sensitivity) Assay Kit (Thermo Fisher Scientific, US). Library quality assessment was performed using an Agilent D5000 ScreenTape System (Agilent, US) in a 4150 TapeStation System (Agilent, US). The library qualification criteria included the presence of a broad peak in the range of 300 bp to 1000 bp, with an average size of 400 bp in the Agilent 4150 TapeStation system, and Qubit concentrations above 2 ng/µl or 10 nmol. Final pooling and sequencing were conducted on a NovaSeq 6000 platform.

## Sequencing and data processing

Sequence data were generated via NovaSeq 6000. Data quality was assessed using the FastQC software^28^. The raw reads were trimmed using trimmomatic^29^. For alignment-based analysis, the cleaned reads were mapped to safflower reference genome version 1^24^ using the bwa mem algorithm^30^. For each sample, the breadth and depth of coverage were determined using the SAM tool package^31^. For alignment-free analysis, a k-mer analysis was conducted. Cleaned reads were subjected to k-mer counting with a k-mer size of 27 nt, and distinct and unique k-mers were identified using jellyfish^32^. The k-mers that appeared only once in the samples were filtered out, as they were likely attributable to sequencing errors. The k-mer-based genetic distance between the 42 samples was measured using Mash^33^. Gene-level k-mer sequence validation was performed by gene presence/absence variation (PAV) in each sample using SGSGeneLoss^34^. Variants from both the sdRAD-seq and ddRAD-seq datasets were called using the bayesian genotype caller and stacks software using “ref_map.pl” pipeline with the remove duplicates option. The “population” pipeline of stacks software^35^ was further utilized to export the detected SNPs in VCF format. Vcftools^36^ was employed to filter the variants, which were subsequently plotted via CMplot^37^. Variant effects were predicted using SnpEff software^38^ and gene annotation of the safflower genome version 1^27^. Principal component analysis (PCA) was performed and plotted using the R package SNPRelate^39^.

## Results

### In Silico simulation

Enzymatic digestion of the reference safflower genome, which was simulated with the four different restriction enzymes, revealed that the Msel enzyme has the highest number of restriction sites in the safflower genome, followed by NlaIII, ApeKI, and EcoRI (Supplementary Fig. S1). In silico digestion showed that the highest total number of DNA fragments was generated by the NlaIII_Msel combination, followed by ApeKI and EcoRI_Msel. After the size selection range of 300 to 700 bp, similar patterns were observed, where NlaIII_Msel resulted in the highest number of fragments, and the lowest number for EcoRI_Msel. A comparison of the fragment size distributions revealed that after size selection, only 5% of the fragments were retained for NlaIII_Msel, whereas for ApeKI and EcoRI_Msel, 12% of total fragments were retained (Supplementary Fig. S2).

## In vitro results

### Sequencing data

sdRAD-seq by ApeKI and ddRAD-seq by NlaIII_Msel and EcoRI_Msel resulted in total raw read counts of 175.46, 424.77 and 332.46 million respectively for the set of 42 diverse safflower accessions. sdRAD-seq by ApeKI resulted in a minimum of 48,978 (Y15) reads and a maximum of 1, 29, 74, and 484 (Y95) reads, with an average of 4.17 million reads per sample. NlaIII_Msel produced 5,664 (L17) lowest and 3,77,84,410 (G54) the highest number of reads, with an average of 10.11 million reads per sample. The EcoRI_Msel combination reported a minimum of 78,854 (X8) and a maximum of 1,91,48,138 (Y16) read counts, with an average of 7.92 million reads per sample (Supplementary Fig. S3).

### Alignment based analysis

For alignment-based analysis, raw reads were trimmed for adapter content, and low-quality reads were aligned to the safflower reference genome. The alignment rate for sdRAD-seq was not uniform among the safflower accessions, varying from 14.21% (N90) to 92.99% (I72). However, in the case of ddRAD-seq, an alignment rate of > 82% was reported. The alignment rates for the NlaIII_Msel and EcoRI_Msel restriction enzyme combination were highly related, and EcoRI_Msel showed a nearly uniform alignment rate for 42 samples (Supplementary Fig. S4). Among the aligned bam files, 46.78% of the ApeKI reads were primary aligned; however, the percentages of the primary aligned NlaIII_Msel and EcoRI_Msel reads were 96.68% and 98.91%, respectively (Supplementary Table S2). Alignment scores for ApeKI, NlaIII_Msel and EcoRI_Msel are 45.56, 106.20 and 119.43 respectively.

### Depth and breadth of coverage

The depth of coverage for each sample was determined using the aligned bam files. The per-sample depth of coverage was lowest for ApeKI, that is, 0.24X with a range from 0.0004X (A82) to 0.6596X (A15). The NlaIII_Msel depth of coverage varied from 0.0005X to 3.9485X, with an average of 1.10X per sample. In the case of EcoRI_Msel, the depth coverage ranged from 0.0091X (X8) to 1.781X (Y16), with a per-sample average of 0.785X (Supplementary Fig. S5 a). The mean breadth coverage for ApeKI was 0.68%, and NlaIII_Msel and EcoRI_Msel had 24.57% and 7.05% coverage breadth, respectively (Supplementary Fig. S5 b).

### Alignment free analysis

#### K-mer (Distinct & Unique) analysis

K-mer frequencies in 42 samples with sdRAD-seq using ApeKI and ddRAD-seq using NlaIII_Msel and EcoRI_Msel were recorded on the basis of distinct and unique K-mer counts. sdRAD-seq by ApeKI resulted distinct k-mers counts ranging from 0.06 million (A15) to 59.91 million (Z13) with an average of 25.92 million per sample (Supplementary Fig. 6a). ddRAD-seq by NlaIII_Msel reported average distinct counts of 172.20 million per sample with a range of 0.34 million (L17) to 397.22 (J77) million k-mers (Supplementary Fig. 6b) whereas for EcoRI_Msel, distinct k-mer frequency varies from 2.39 million (X8) to 141.22 million (Y16), with an average of 76.20 million per sample (Supplementary Fig. 6c). Unique K-mers comparison shown that sdRAD-seq by ApeKI generated more common K-mers (88.22%) than the unique K-mers (11.78%) for 42 safflower samples. However, ddRAD-seq by NlaIII_Msel captured 46.40% unique K-mers and 58.12% EcoRI_Msel.

### K-mer sketching based on genetic distance

**Fig. 1:**
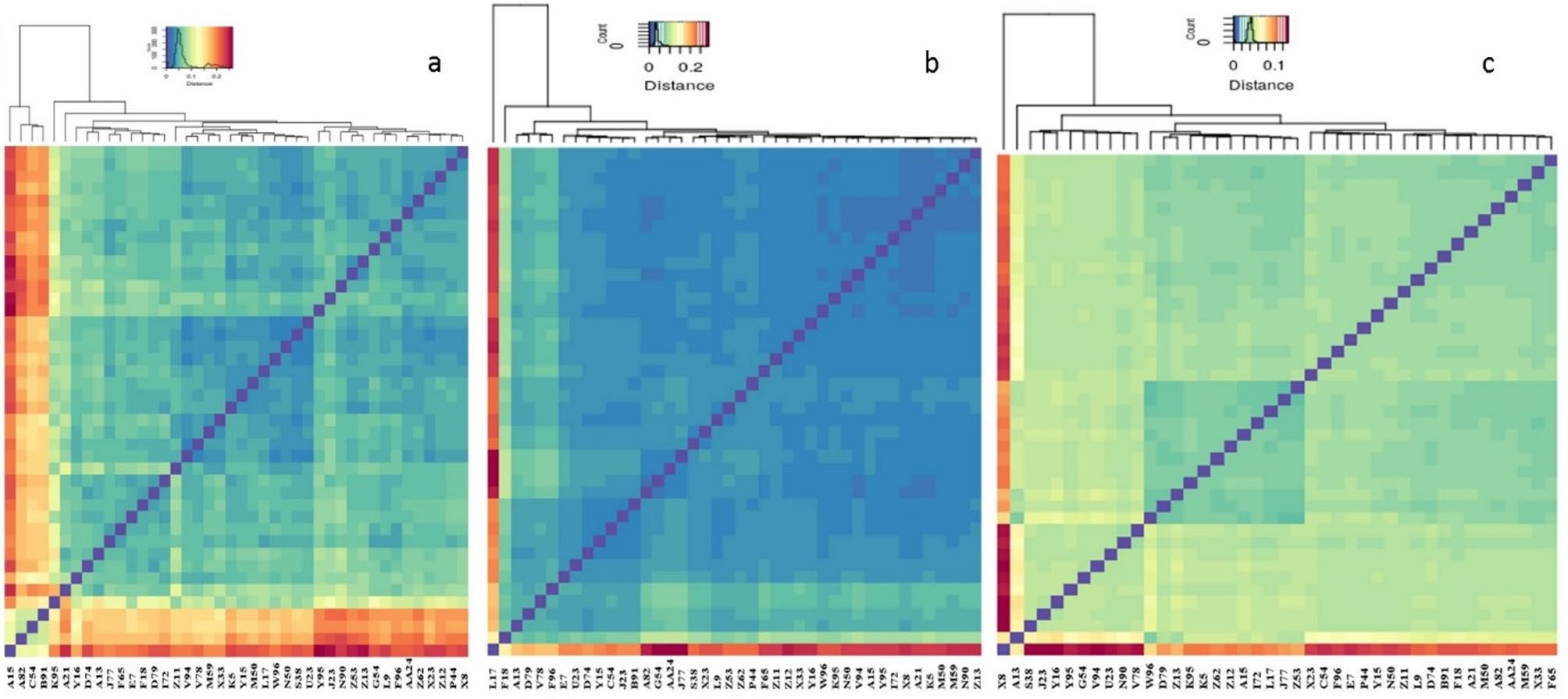
Mash based genetic distance, (**a**) ApeKI, (**b**) NlaIII_Msel and (**c**) EcoRI_Msel.

The k-mer-based genetic distance between each pair of samples was calculated and sketched to create a reduced representation while unique or low-copy k-mers were removed (minimum copies of each k-mer to filter unique k-mers were specified as 2), since these k-mers are likely be the result of sequencing errors. Genetic distance measures variation between accessions. For the ApeKI k-mer-based genetic distance between each pair of samples, A15, A82, C54, B91, and K95 presented high genetic distances (Fig. 1a). L17 and F18 showed high genetic distance for NlaIII_Msel (Fig. 1b) and EcoRI_Msel (X8 and A13), whereas samples with a low number of k-mers reflected high genetic distance (Fig. 1c). In addition to the aforementioned samples, the maximum variability in ApeKI was 0.19%. For NlaIII_Msel, apart from the L17 and F18 samples, the maximum variability reported was 0.19%. Similarly, for EcoRI_Msel, except for samples X8 and A13, the maximum variability reported was 0.13%.

### Gene level validation (distribution and annotation) of k-mers

The sdRAD-seq (ApeKI) and ddRAD-seq (NlaIII_Msel and EcoRI_Msel) k-mer sequence data were assessed for gene variability in 42 safflower samples, where genes covered in all samples by k-mer data were considered core genes and genes absent in any of the 42 safflower samples were considered variable genes. sdRAD-seq by ApeKI identified 16 core and 17,393 variable genes. Sixteen core genes were able to cover chromosomes 1,2,3,6,10 and chromosome 12, with 3, 4,1,1,5 and 2 genes, respectively. ddRAD-seq via NlaIII_Msel restriction enzyme combination was able to capture 258 core genes and 32,998 variable genes. With 19, 45, 23, 25, 13, 26, 15, 15,6, 31, 18, and 22 genes corresponding to chromosomes 1 through 12,258 core genes were able to cover all the chromosomes. ddRAD-seq via EcoRI_Msel described 946 core and 30,217 variable genes in the set of 42 samples. All 12 chromosomes were covered by 946 core genes, which included 72, 93, 89, 83, 66, 105, 60, 66, 67, 105,68 and 72 genes for 12 chromosomes (Supplementary Fig. S7 a). The gene ontology of sdRAD-seq (ApeKI) core genes involved in four molecular functions and two biological processes associated with three cellular components and were mainly related to photosynthesis (photosystem II reaction center protein A and B and photosynthetic electron transfer A) and ATP metabolic processes (proton transmembrane transporter activity) (Supplementary Fig. S7 b). The core genes of ddRAD-seq by NlaIII_Msel engaged in 41 molecular functions and 30 biological processes and were found in 16 cellular components (Supplementary Fig. S7 c), mainly from biological processes such as photosynthesis, transcription, translation, and DNA integration, whereas core genes from ddRAD-seq through EcoRI_Msel had 66 molecular functions, 49 biological processes, and 23 cellular components and were similarly involved in biological processes such as photosynthesis, transcription, translation, DNA integration, and oxidation reduction processes (Supplementary Fig. S7 d).

### Variant calling

Variant calling from the aligned reads for sdRAD-seq (ApeKI) and ddRAD-seq (NlaIII_Msel and EcoRI_Msel) resulted in 6,721, 173,212, and 221,805 SNPs, respectively. Subsequently, SNPs were filtered using minor allele frequency (MAF) 5% and genotype coverage of 80%, which retained 289, 8,292, and 37,226 SNPs from the sdRAD-seq (ApeKI) and ddRAD-seq (NlaIII_Msel and EcoRI_Msel) datasets, respectively (Table 2). SNP statistics based on total aligned reads from the sequenced data indicated that ddRAD-seq generated more SNPs with fewer reads per SNP per sample, and the enzyme combination EcoRI_Msel exhibited superior performance compared with NlaIII_Msel. SNP analysis to assess the extent of missing data in SNP genotyping revealed that ddRAD-seq performed better than sdRAD-seq. EcoRI_Msel restriction enzyme combination produced more SNPs i.e. approximately 28% higher than NlaIII_Msel with fewer missing observations (Table 2), reflecting its genotype coverage potential. A higher number of SNP and larger sample coverage will give better representation of populations genetic diversity.

**Table 2:** SNP statistic for ApeKI and two restriction enzyme combinations (NlaIII_Msel and EcoRI_Msel).

Statistic	ApeKI	NlaIII_Msel	EcoRI_Msel
Total SNPs	6,721	1,73,212	2,21,805
Total aligned reads in bam files	10,35,40,252	47,18,49,895	33,83,30,252
Mean aligned reads/SNP	15,405	2,724	1,525
Mean aligned reads/SNP/sample	366	65	36
SNP with MAF 5% and genotype coverage 80%	289	8,292	37,226
SNP with MAF 5% and genotype coverage 100%	-	1	306

### Polymorphic information content (PIC), SNP distribution & annotation for ddRAD-seq data

**Fig. 2:**
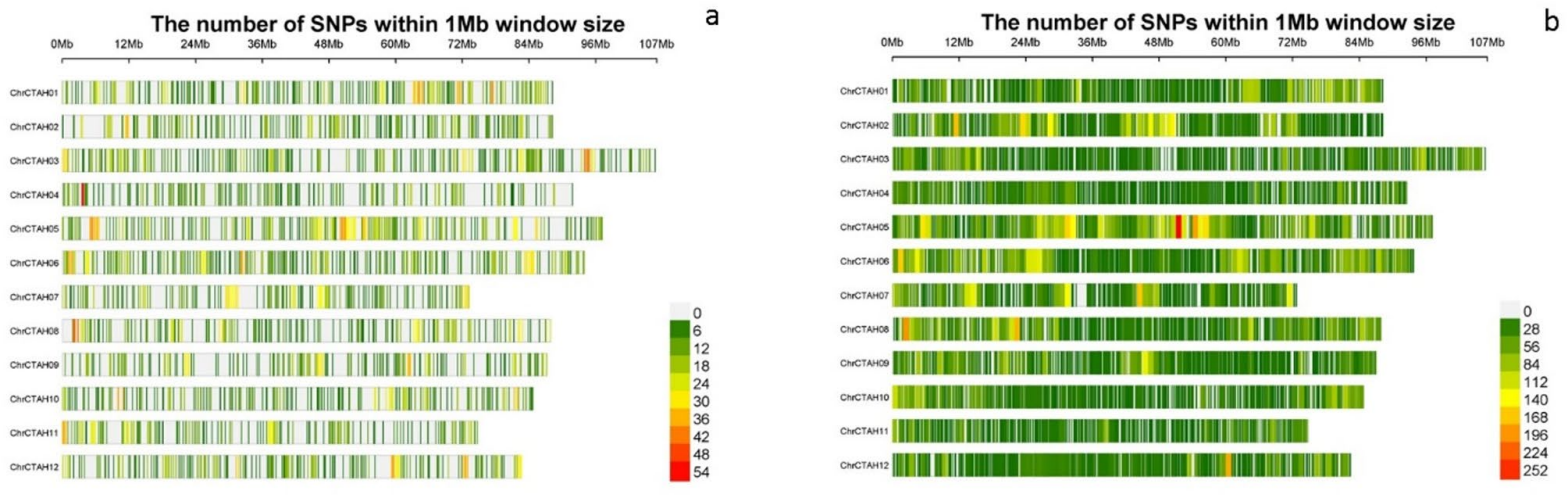
Density of SNPs along chromosomes, (**a**) NlaIII_Msel and (**b**) EcoRI_Msel.

SNP statistics revealed that ddRAD-seq generated a higher number of SNPs, PIC analysis and annotation was performed exclusively for the ddRAD-seq data. In ddRAD-seq, NlaIII_Msel and EcoRI_Msel produced 8,292 and 37,226 SNPs, respectively. In both the enzymes combinations, the average PIC content was 0.30, with a range from 0.09 to 0.38. For NlaIII_Msel, the variant rate was 1 variant per 127,486 bases, with the highest number of SNPs on chromosome 5 (986 SNPs) and the lowest number of SNPs observed on chromosome 11 (536 SNPs) (Fig. 2a, Supplementary Table S3). The majority of the SNPs (73.94%) were located in intergenic regions, of which 13.015% were upstream and 14.441% were found in the downstream region of an open reading frame (ORF). A limited percentage of SNPs were observed in the intronic (16.121%) and exon regions (7.47%) compared with the 3’UTR (1.074%) and 5’UTR (0.936%) (Supplementary Fig. 8S a). Based on nucleotide substitutions (transitions (Ts) and transversions (Tv)), 5,589 transitions and 2,703 transversions were detected with a transition/transversion (Ts/Tv) ratio of 2.06. The transition frequency was highest for G/A, and the transversion frequency was highest for A/T (Supplementary Fig. 8S b). With respect to EcoRI_Msel, the variant rate was one variant per 28,397 bases, with chromosome 5 having the largest number of SNPs (5,515 SNPs) and chromosome 11 having the lowest number of SNPs (1,717 SNPs) (Fig. 2b, Supplementary Table S2). The highest concentration of SNPs was positioned in intergenic regions, comprising 67.15%; of these, 13.107% were upstream and 15.282% were downstream of an open reading frame (ORF). A moderate proportion of SNPs was observed when the intronic (20.485%) and exon regions (9.237%) were compared with the 3’UTR (1.262%) and 5’UTR (1.315%) (Supplementary Fig. S8 a). A total of 23,496 transitions and 13,730 transversions were identified on the basis of nucleotide substitutions (transitions (Ts) and transversions (Tv)). The ratio of transitions to transversions (Ts/Tv) is 1.71. G/A presented the highest transition frequency, whereas C/A presented the highest transversion frequency (Supplementary Fig. S8 b). Nevertheless, similarity was observed in the chromosome distribution pattern of SNPs in both enzyme combinations of ddRAD-seq and in the investigation of the structural and functional relevance of SNPs, which also followed a comparable trend.

### Genetic diversity and relationships

**Fig. 3:**
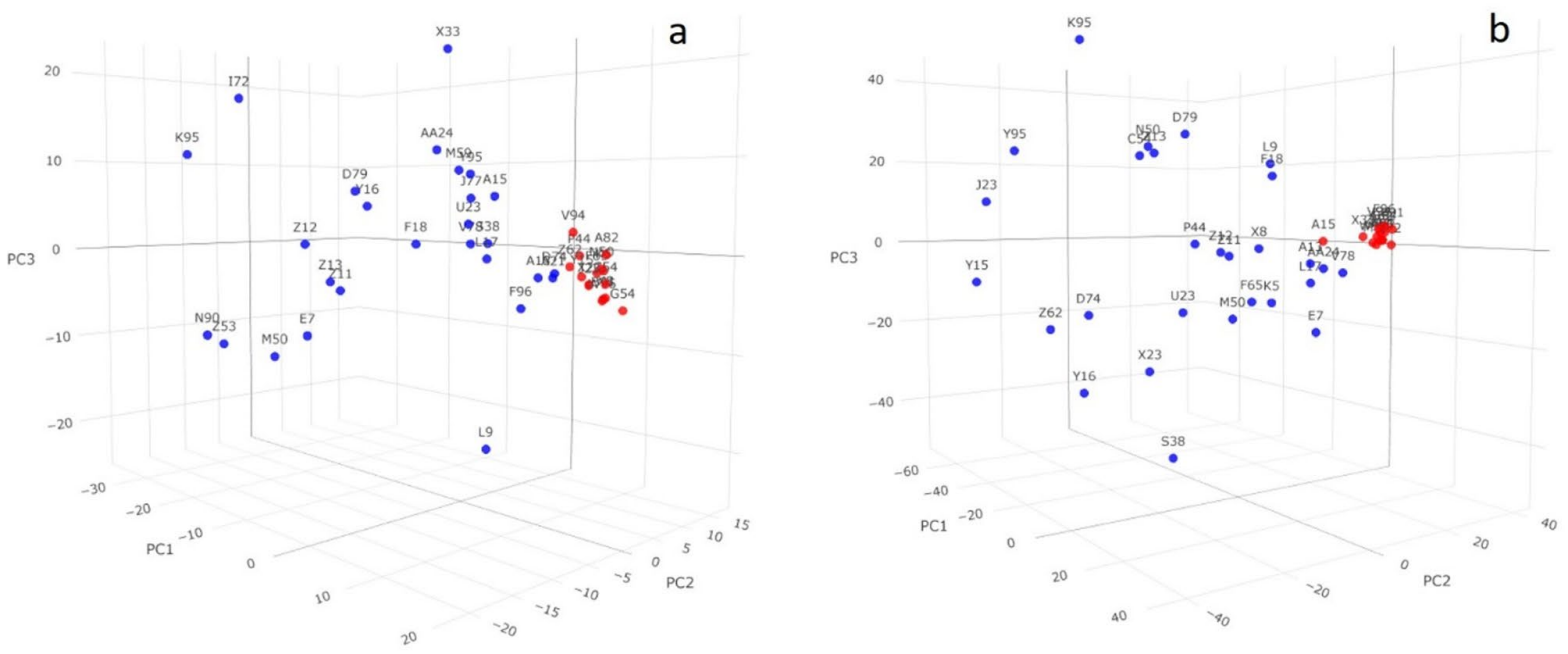
Scatter plot of three principal components (PCs) for ddRAD, (**a**) NlaIII_Msel, (**b**) EcoRI_Msel. In both enzyme combinations, accessions that are clustered together are highlighted as red dots.

**Fig. 4:**
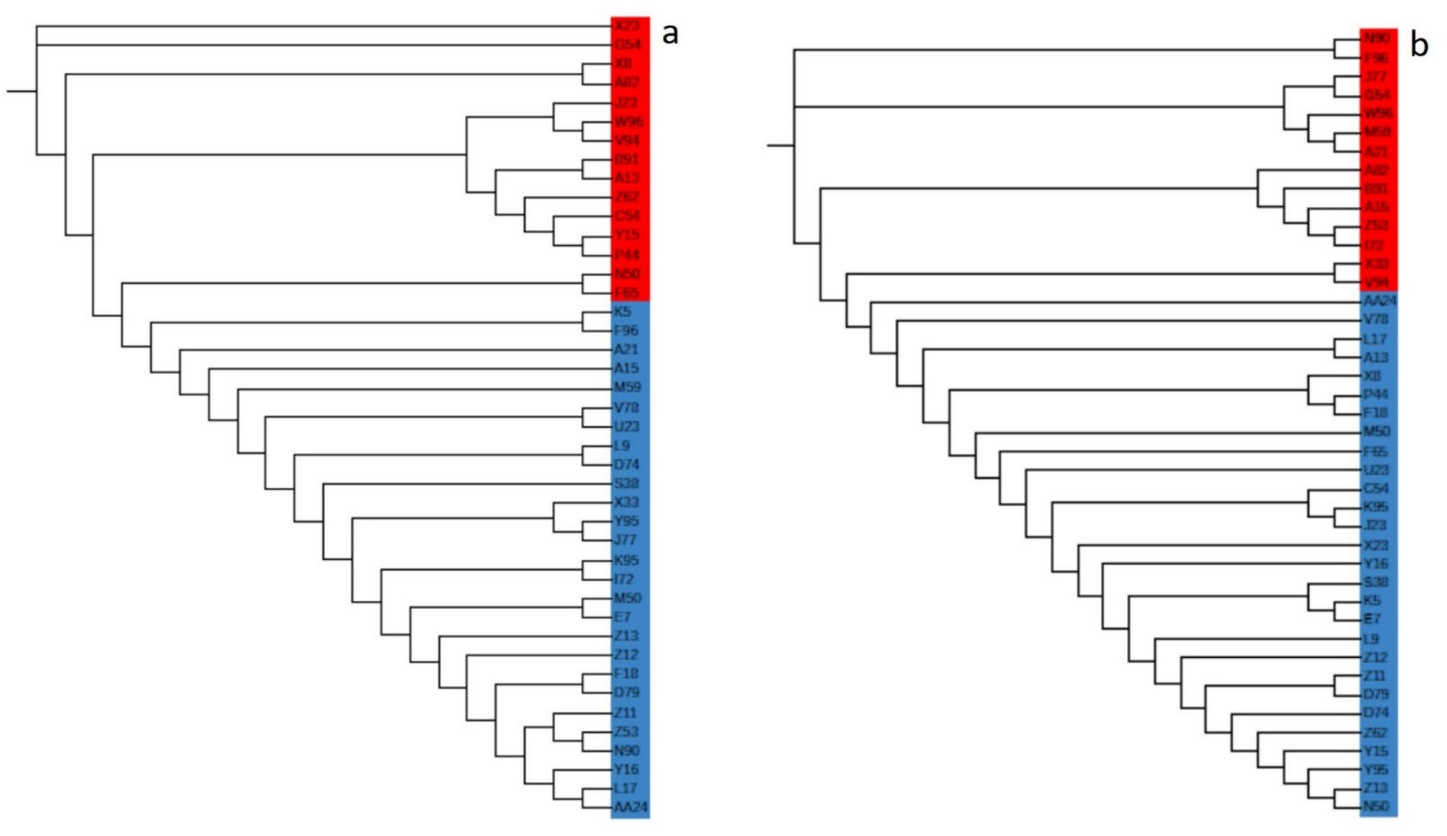
Neighbor-Joining clustering for 42 safflower accessions, **a**) NlaIII_MseI, (**b**) EcoRI_MseI. Accessions that are clustered together in the PCA plots (highlighted in red) are also marked in red here, demonstrating consistent groupings across these two analyses.

The genetic parameters (observed and expected heterozygosity, homozygosity, and gene diversity) were investigated for 8,292 and 37,226 SNP markers recorded by NlaIII_Msel and EcoRI_Msel, respectively, for the 42 safflower accessions. The SNP data for NlaIII_Msel revealed an average gene diversity of 0.38, ranging from 0.38 to 0.39, with the results indicating that the highest gene diversity (0.39) was observed for accessions (B91, F96, X23, and A13) from India. Additionally, the observed heterozygosity (Ho) ranged from 0 to 0.06, with an average of 0.04. With EcoRI_Msel, an average gene diversity of 0.29 was recorded, with a range from 0.29 to 0.30. The highest gene diversity (0.30) was observed for 12 accessions, of which five (C54, D79, F18, L9, and N90) originated from India, four (D74, K95, P44, and S38) from the USA, and one each from Italy (K5) and Afghanistan (Z13). The observed heterozygosity (Ho) in this case ranged from 0.01 to 0.04, with an average of 0.02. For both restriction enzyme combinations for ddRAD, the expected heterozygosity (He) was higher than the observed heterozygosity (Ho) (Table S4). The principal component analysis (PCA) for ddRAD-seq indicated that in NlaIII_Msel, the first principal component (PC) accounted for 19.70% of the total variation (Fig. 3a), whereas in EcoRI_Msel, the PC1 accounted for 20.54% of the total variation (Fig. 3b). The cumulative variation in the first three principal components for NlaIII_Msel and EcoRI_Msel was 30.29% and 33.98%, respectively. Phylogenetic analysis utilizing the Neighbor-Joining method revealed the clustering of 42 accessions into three primary groups in both cases. For NlaIII_Msel, the first two clusters each comprised a single accession (X23 and G54), whereas the remaining 40 accessions were grouped into a single cluster. Conversely, for EcoRI_Msel, the first cluster contained two accessions (N90, F96), the second cluster included five accessions (J77, G54, W96, M59, A21), and the remaining 35 accessions formed a separate, larger cluster. (Fig. 4).

## Discussion

Genotyping-by-sequencing (GBS) approaches are designed to sequence genomes to explore genetic variations and perform genome-wide association mapping^16^. The reduced representation methods of GBS offer the advantages of being expeditious, efficient, and economical for genome-wide genetic variation analysis and association studies^13,40^ via restriction enzyme (RE)-assisted genome reduction^41,42^ and SNP calls with or without a sequenced genome^18,43^. Selecting the appropriate restriction enzyme is a crucial initial step in the development of a GBS protocol^44^. The high-quality genome assembly of safflower reported^27^ could serve as a valuable reference for such ddRAD studies, enabling more precise genetic analyses and potentially uncovering novel aspects of safflower diversity and evolution. This suggests that high-throughput sequencing approaches like ddRAD could potentially provide even more comprehensive insights into safflower genetics. Its application could potentially overcome limitations of previous marker systems and provide more detailed insights into safflower genetics.

The simulated prediction of safflower genome complexity reduction utilized a combination of rare cutter enzymes (NlaIII and EcoRI) and frequent cutter enzymes (Msel), and a comparison of the results with a single restriction reaction (ApeKI), revealed a higher number of fragments with NlaIII_Msel ranging between 300 and 700 bp. However, different restriction enzyme combinations have been employed in previous studies, depending on the experimental requirements and analyses. For instance, GBS-based phylogenetic analyses of *C. tinctorius* together with wild species of *Carthamus*, that is, *C. boissieri*, C. *glaucus*, *C. lanatus*, *C. oxyacantha*, *C. palaestinus*, and *C. tenuis*, utilized the PstI_MspI restriction enzyme combination and obtained 1,582 loci for a dataset of 60 individuals^17^. To study the diversity, population structure, and marker–trait associations of 94 safflower accessions from the United States Department of Agriculture (USDA) via DArTseq, the PstI–MseI restriction enzyme combination was utilized^45^. In our, in vitro experiments, the overall sequence read representation was lower in the sdRAD-seq (ApeKI) data than in the ddRAD-seq data. The NlaIII_Msel enzyme combination obtained the highest average number of raw reads per sample (> 10 million), followed by EcoRI_Msel, with more than 7 million raw reads per sample. ddRAD-seq demonstrated superior performance compared with sdRAD-seq under actual experimental conditions. ApeKI (G/CWGC), used for sdRAD-seq, is a GC-rich sequence recognition enzyme that cannot cleave AT-rich DNA regions. Additionally, ApeKI exhibits partial methylation sensitivity, which affects its digestion under wet laboratory conditions, resulting in outcomes that differ from those of in silico analysis. Similar results were observed in soybean, where ApeKI produced fewer fragments in actual experiments than in silico simulations did^44^. The frequent cutter MseI (T/TAA) used for ddRAD-seq recognizes AT-rich sequences and is not sensitive to DNA cytosine methylation (Dcm), deoxyadenosine methylation (Dam), or CpG methylation. Therefore, it is not influenced by DNA methylation^46^.

Based on the alignment percentage, the performance of the tested restriction enzymes demonstrated that the overall performance of EcoRI_Msel exhibited a similar trend across 42 samples, whereas for NlaIII_Msel and ApeKI, the alignment percentage varied across a broad scale. For alignment-based analysis, measuring the depth of sequencing coverage is critical for genomic analyses. NlaIII_Msel reported a higher depth of coverage because the coverage parameter was directly influenced by the read number, indicating that a higher read number corresponds to a higher depth of coverage^47^. To expedite genotyping and minimize reference bias, an alignment-free algorithm was employed^48^ for genome assembly and comparison. The most widely used alignment-free method is based on the k-mer count, where sequence information is transformed into numerical values such as the k-mer count, and their frequencies are further utilized to calculate distances between samples^49^. Our results indicate that ddRAD-seq performed better than sdRAD-seq in producing both distinct and unique k-mers. Gene-level validation of the k-mer data revealed that ddRAD-seq by EcoRI_Msel was able to cover more genes, that is, 946 genes in 42 samples, whereas ddRAD-seq by NlaIII_Msel covered only 258 genes in all the samples.

The improvement in SNP genotyping through restriction enzyme combinations can be assessed by SNP analysis with respect to the extent of missing data. In our study, NlaIII_Msel covered a smaller proportion of the genome than EcoRI_Msel (Fig. 2). SNP statistics revealed that EcoRI_Msel displayed more SNPs, with 80% genotype coverage and no missing observations. The He represent gene diversity and Ho represents observed heterozygosity. This helps in understanding the population structure and genetic diversity of population. In safflower using the enzyme combination we could be able to derive higher gene diversity (He) and lower heterozygosity (Ho).

The first three principal components in the PCA explained 30.29% and 33.98% of the total genetic variation for samples sequenced by ddRAD-Seq using the NlaIII_Msel and EcoRI_Msel restriction enzymes, respectively. PCA results from NlaIII_Msel showed a slightly less distinct separation of accessions, as they were tightly grouped, whereas the PCA findings from EcoRI_Msel could be more readily utilized to identify duplicates in samples. Principal Coordinates Analysis (PCoA) and phylogenetic analysis for both enzyme combinations exhibit a strong correlation. For NlaIII_Msel, accessions highlighted in red on the phylogenetic tree were consistently clustered together in the PCoA plot. These clumped accessions represent samples from diverse geographical regions, including India, Ethiopia, the USA, Germany, and Australia. In the case of EcoRI_Msel, the accessions marked in red on the phylogenetic tree also appeared to cluster together in the PCoA analysis, with these accessions being exclusively from India and the USA. Notably, EcoRI_Msel demonstrated a greater ability to distinguish accessions based on their geographical origins, further emphasizing its utility in differentiating safflower accessions from distinct regions. These findings demonstrate that among the two different approaches of reduced representation, the ddRADseq method generated significantly more variants among the accessions compared to sdRADseq. The enzyme combination EcoRI_MseI generated variants with 5% MAF more effectively than NlaIII_MseI, demonstrating the potential of ddRAD with the restriction enzyme combination EcoRI_MseI for advanced genome sampling and SNP genotyping in safflower for GBS applications.

Overall, Double-digest RAD sequencing (ddRAD-seq) offers several advantages over single-digest RAD sequencing (sdRAD-seq) for plant genotyping. ddRAD-seq utilizes two restriction enzymes, providing more flexibility and balance in genomic loci analysis. This approach allows for the examination of hundreds of individuals, making it particularly suitable for large-scale genetic and genomic studies in plants. ddRAD-seq technique that involves the production of multiplexed libraries by enzymatically digesting whole genomic DNA and then binding specific adapters, resulting in reduced representation libraries. By restricting the portion of the genome sequenced, ddRAD-seq generates a comprehensive set of SNP markers that can be used to precisely assess genetic diversity and population structure in large germplasm collections. The use of double restriction digestion also facilitates paired-end sequencing of identical loci across multiple samples, enhancing read mapping accuracy compared to sdRAD-seq.

While ddRAD-seq has been successfully applied to various diverse crops, such as tomato^50^ and seasame^23^, its use in safflower germplasm investigations has been relatively scarce. The present study will provide insight in to genotyping of germplasm collection conserved at Indian National Gene Bank for developing core collection harbouring maximum genetic diversity to be used as parental lines for varietal development program. The robust cost effective ddRAD markers with suitable enzyme combinations will provides maximum SNPs with wide genomic coverage for precise development of core collection. This method is well-suited for large-scale genotyping and can be implemented in extensive breeding programs. However, its effectiveness depends on the number of reads generated and the suitable combination of restriction enzymes, which maximizes the reads and thus leads to effective coverage for discovering genomic regions associated with various traits.

## Conclusion

This study compared the sdRAD-seq and ddRAD-seq methods for SNP discovery and genetic diversity assessment in safflower (*Carthamus tinctorius* L.) using in silico and in vitro approaches. Three restriction enzyme combinations (ApeKI, NlaIII_Msel, and EcoRI_Msel) were tested in 42 diverse safflower accessions. In silico testing revealed that NlaIII_Msel generated the highest number of DNA fragments. The in vitro results demonstrated that ddRAD-seq outperformed sdRAD-seq in read count, alignment rate, coverage, and SNP detection. Alignment-free k-mer analysis confirmed the superiority of ddRAD-seq, with EcoRI_Msel identifying more core genes than other combinations. EcoRI_Msel captured more SNPs with fewer missing observations. Principal component analysis using ddRAD-seq data explained substantial genetic variation. The study concluded that ddRAD-seq with EcoRI_Msel is the most suitable genotyping-by-sequencing approach for genome sampling and SNP genotyping in safflower.

## Electronic supplementary material

Below is the link to the electronic supplementary material.

Supplementary Material 1

Supplementary Material 2

Supplementary Material 3

Supplementary Material 4

Supplementary Material 5

Supplementary Material 6

Supplementary Material 7

Supplementary Material 8

Supplementary Material 9

Supplementary Material 10

Supplementary Material 11

Supplementary Material 12

## Data Availability

Sequence data that support the findings of this study have been deposited in the Indian Biological Data Centre (IBDC) (https://ibdc.dbtindia.gov.in/inda/completeStudyDetailsById?studyid=INRP000244) Accession No. INRP000244 and INSDC Project Accession No. ERP167403 (https://www.ebi.ac.uk/ena/browser/view/PRJEB83828)/ PRJEB83828 (https://www.ncbi.nlm.nih.gov/bioproject/PRJEB83828).
