# Supplementary material for: Comparative analysis of RAD-seq methods for SNP discovery and genetic diversity assessment in oil seed crop safflower
Source: Sci Rep. 2025 Jul 2;15:22600. doi: 10.1038/s41598-025-06706-2 (PMC12217066; doi:10.1038/s41598-025-06706-2)
Supplement: Supplementary file 2 — Supplementary Material 2 [file 41598_2025_6706_MOESM2_ESM.docx]

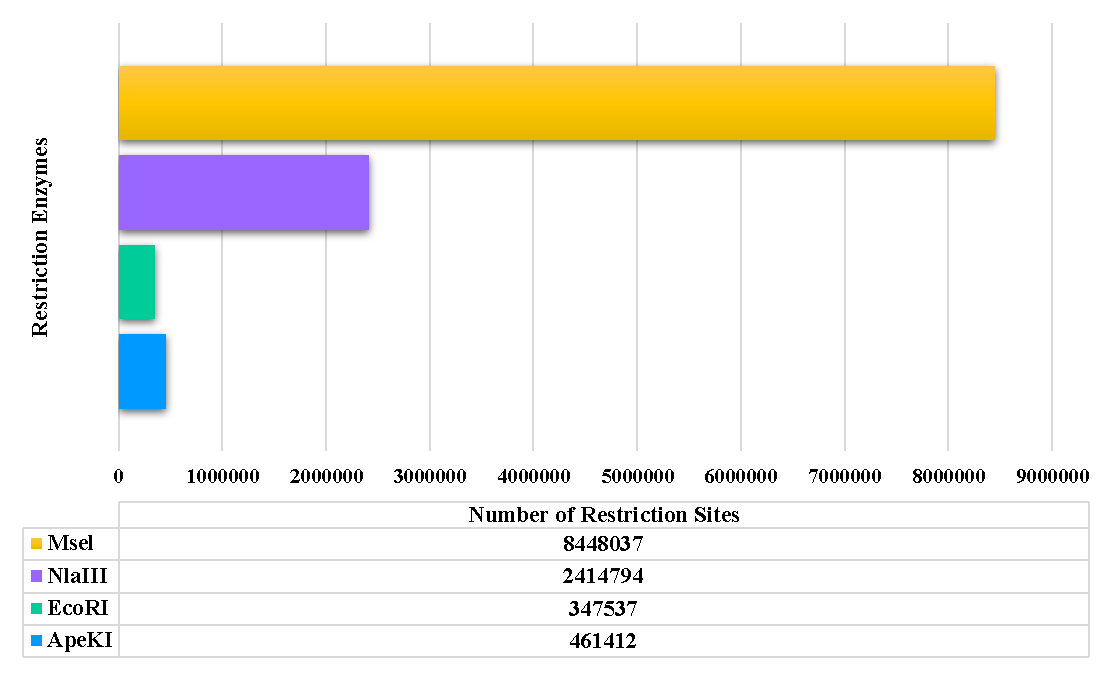


**Fig. S1** Frequency of restriction sites in the safflower genome with corresponding enzymes.
MseI, a frequent cutter, is reported to have the highest number of restriction sites in the safflower genome, with 8,448,037 sites. In contrast, for rare cutters, the length of the enzyme’s recognition sequence plays a significant role in the number of sites detected. NlaIII, which recognizes a 4-base pair sequence, have statistically more frequent sites than ApeKI, which recognizes a 5-base pair sequence, and EcoRI, which recognizes a 6-base pair sequence. This difference in recognition sequence length explains the variation in the number of restriction sites observed for each enzyme.
