# Supplementary figures and images for: Comparative analysis of RAD-seq methods for SNP discovery and genetic diversity assessment in oil seed crop safflower
Source: Sci Rep. 2025 Jul 2;15:22600. doi: 10.1038/s41598-025-06706-2 (PMC12217066; doi:10.1038/s41598-025-06706-2)

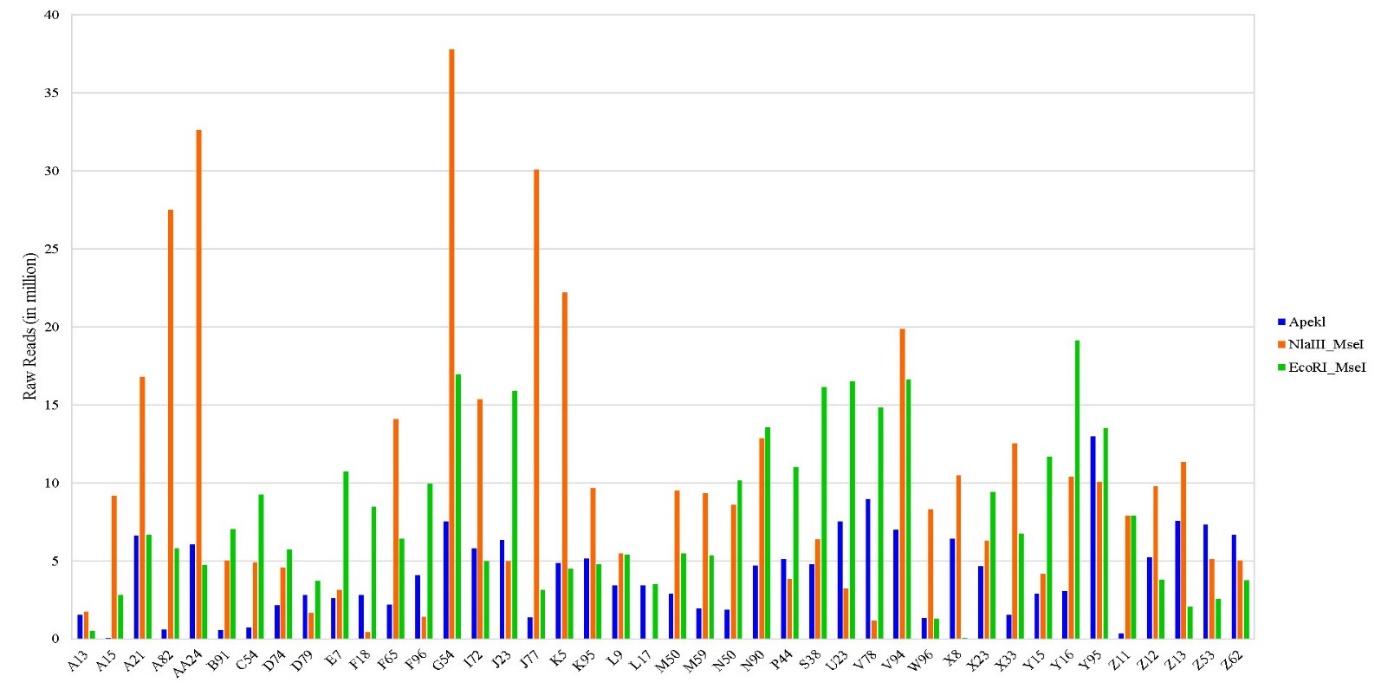


**Fig. S3** Distribution pattern of raw reads per sample using different restriction enzymes

Supplement: Supplementary file 3 — Supplementary Material 3 [file 41598_2025_6706_MOESM3_ESM.docx]

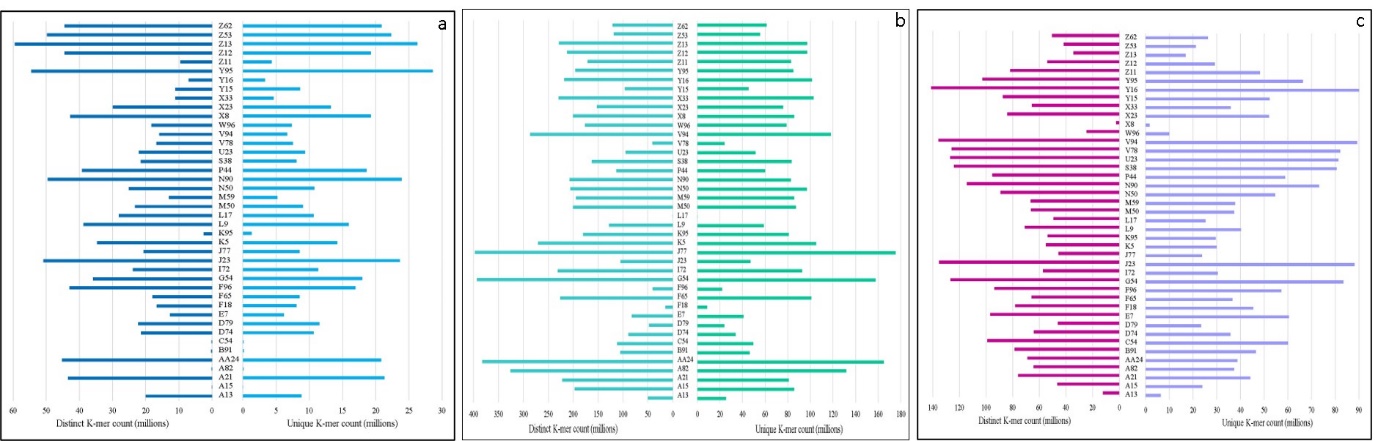


**Fig. S6** Distinct and unique k-mer count, a) ApeKI, b) NlaIII_Msel and c) EcoRI_Msel

Supplement: Supplementary file 5 — Supplementary Material 5 [file 41598_2025_6706_MOESM5_ESM.docx]

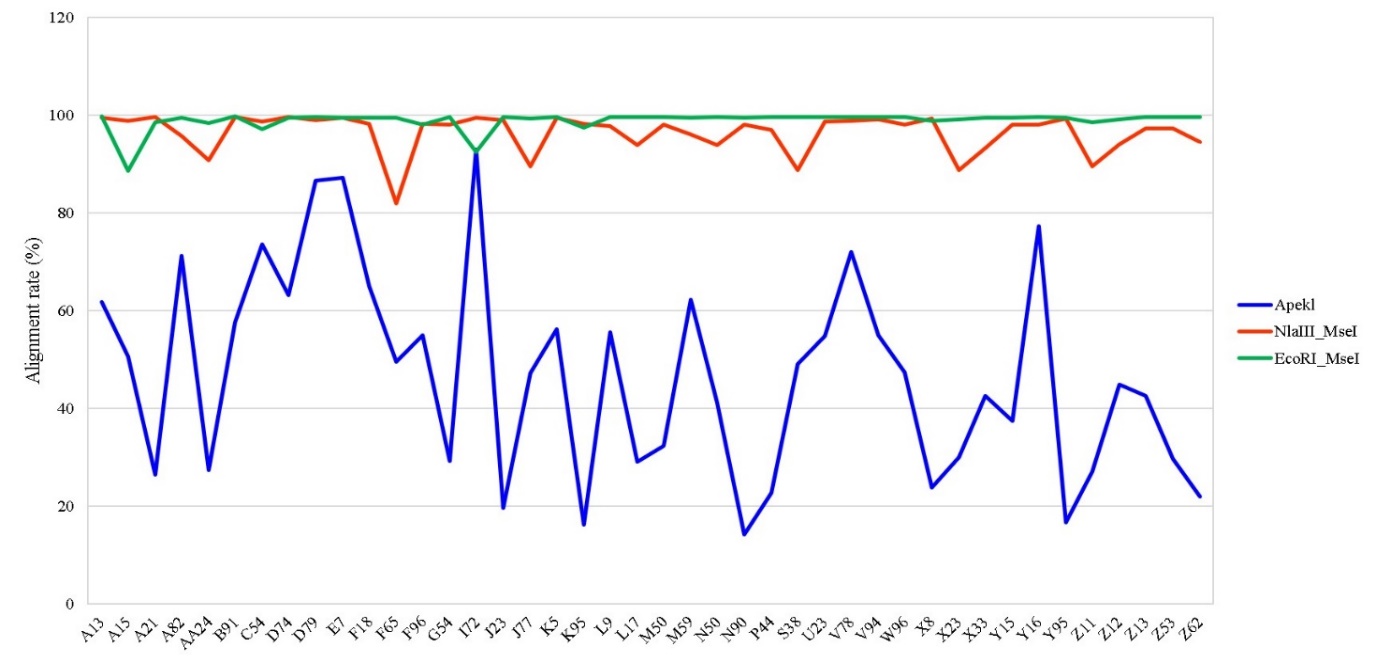


**Fig. S4** Alignment rate of trimmed data using different restriction enzymes

Supplement: Supplementary file 7 — Supplementary Material 7 [file 41598_2025_6706_MOESM7_ESM.docx]

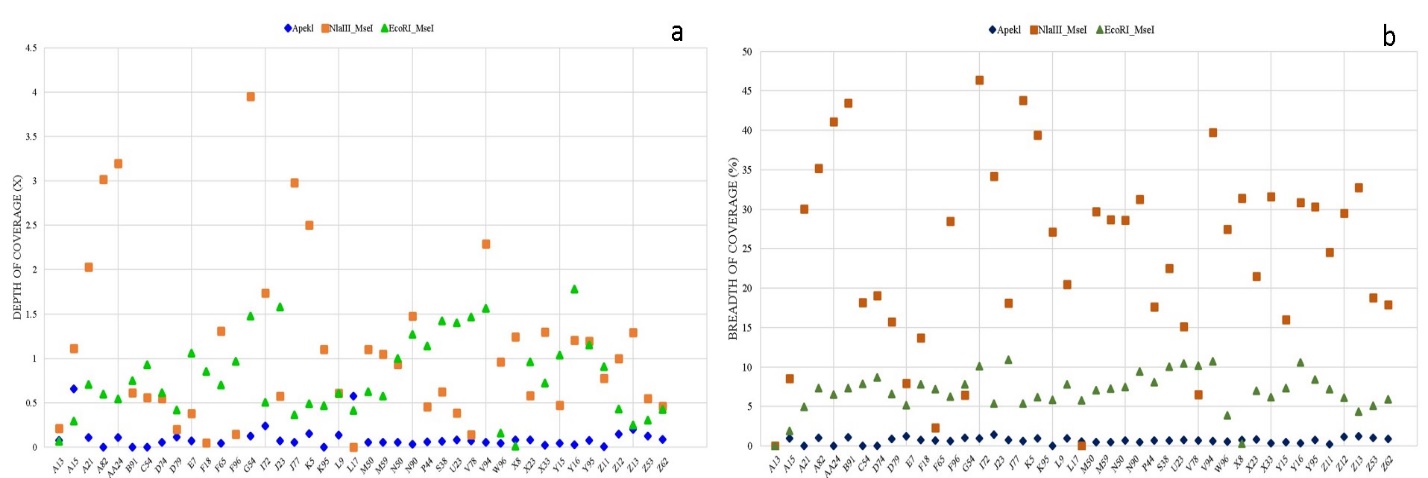


**Fig. S5** a) Depth of coverage (X) b) Breadth of coverage (%)

Supplement: Supplementary file 10 — Supplementary Material 10 [file 41598_2025_6706_MOESM10_ESM.docx]
