# Supplementary material for: Comparative analysis of RAD-seq methods for SNP discovery and genetic diversity assessment in oil seed crop safflower
Source: Sci Rep. 2025 Jul 2;15:22600. doi: 10.1038/s41598-025-06706-2 (PMC12217066; doi:10.1038/s41598-025-06706-2)
Supplement: Supplementary file 4 — Supplementary Material 4 [file 41598_2025_6706_MOESM4_ESM.docx]

**Table S1 Details of the safflower accessions used in the study**

| **Sample code** | **Accession Number** | **Bio status** | **Source** |
| --- | --- | --- | --- |
| A13 | IC 253214 | Landrace | Andhra Pradesh |
| A15 | IC 0218905 | Landrace | Haryana |
| A21 | IC 249559 | Wild | Telangana |
| A82 | IC 442789 | Breeding/Adv. breeding line | Maharashtra |
| AA24 | EC 0137296 | Genetic stock | USA |
| B91 | IC 443015 | Landrace | Madhya Pradesh |
| C54 | IC 537575 | Wild | Uttar Pradesh |
| D74 | EC 181159 | Landrace | USA |
| D79 | EC 36340 | Landrace | Rajasthan |
| E7 | IC 442697 | Breeding/Adv. breeding line | Maharashtra |
| F18 | IC 442738 | Breeding/Adv. breeding line | Maharashtra |
| F65 | IC 500081 | Breeders Line | Unknown |
| F96 | IC 443033 | Landrace | Madhya Pradesh |
| G54 | IC 442846 | Breeding/Adv. breeding line | Maharashtra |
| I72 | IC 249561 | Wild | Telangana |
| J23 | EC0303714 | Landrace/Traditional cultivar | Ethiopia |
| J77 | EC 0337639 | Landrace | USA |
| K5 | EC 0151809 | Others/Unknown | Italy |
| K95 | IC 0398320 | Landrace/Traditional cultivar | USA |
| L9 | IC 0031373 | Landrace | Punjab |
| L17 | IC 0042470 | Landrace | Gujarat |
| M50 | IC 0218951 | Landrace | Himachal Pradesh |
| M59 | EC 181652 | Landrace | USA |
| N50 | EC 191790 | Landrace | Australia |
| N90 | IC 302753 | Genetic stock | Maharashtra |
| P44 | EC 0398080 | Landrace/Traditional cultivar | USA |
| S38 | EC 542467 | Improved cultivar | USA |
| U23 | EC 147747 | Released variety | USA |
| V78 | EC 181159 | Landrace | USA |
| V94 | EC 181574 | Landrace | USA |
| W96 | EC 181308 | Genetic stock | USA |
| X8 | EC 181296 | Landrace | USA |
| X23 | EC 0042478 | Landrace | Gujarat |
| X33 | IC 0500009 | Breeding/Adv. breeding line | Unknown |
| Y15 | EC 0033271 | Others/Unknown | Germany |
| Y16 | EC 0137343 | Others/Unknown | Italy |
| Y95 | IC 0147753 | Released variety | Maharashtra |
| Z11 | EC 0321524 | Others/Unknown | Mexico |
| Z12 | EC 0321517 | Others/Unknown | Israel |
| Z13 | EC 0321537 | Others/Unknown | Afghanistan |
| Z53 | IC 0253155 | Landrace | Maharashtra |
| Z62 | EC0398060 | Others/Unknown | USA |
