# Supplementary material for: Comparative analysis of RAD-seq methods for SNP discovery and genetic diversity assessment in oil seed crop safflower
Source: Sci Rep. 2025 Jul 2;15:22600. doi: 10.1038/s41598-025-06706-2 (PMC12217066; doi:10.1038/s41598-025-06706-2)
Supplement: Supplementary file 8 — Supplementary Material 8 [file 41598_2025_6706_MOESM8_ESM.docx]

**Table S3 SNP count in sdRAD-seq and ddRAD-seq datasets**

|  | **sdRAD (Apekl)** | | **ddRAD (NlaIII_MseI)** | | | | **ddRAD (EcoRI_MseI)** | |
| --- | --- | --- | --- | --- | --- | --- | --- | --- |
| **Chromosome** | **Raw SNPs** | **80% genotype coverage**  **& 0.05 MAF** | | **Raw SNPs** | **80% genotype coverage**  **& 0.05 MAF** | **Raw SNPs** | | **80% genotype coverage**  **& 0.05 MAF** |
| chr1 | 538 | 15 | | 16698 | 809 | 18451 | | 3127 |
| chr2 | 343 | 15 | | 14003 | 600 | 19792 | | 3805 |
| chr3 | 492 | 24 | | 16011 | 826 | 20474 | | 2831 |
| chr4 | 294 | 10 | | 11567 | 564 | 18863 | | 2265 |
| chr5 | 918 | 38 | | 26058 | 986 | 27553 | | 5515 |
| chr6 | 863 | 32 | | 18557 | 862 | 21617 | | 4315 |
| chr7 | 453 | 17 | | 11773 | 576 | 13706 | | 2578 |
| chr8 | 870 | 39 | | 13630 | 644 | 18332 | | 3457 |
| chr9 | 609 | 48 | | 12252 | 602 | 16851 | | 2681 |
| chr10 | 501 | 18 | | 12086 | 615 | 16132 | | 2525 |
| chr11 | 462 | 21 | | 9606 | 536 | 13426 | | 1717 |
| chr12 | 378 | 12 | | 10971 | 672 | 16608 | | 2410 |
