# Supplementary material for: Comparative analysis of RAD-seq methods for SNP discovery and genetic diversity assessment in oil seed crop safflower
Source: Sci Rep. 2025 Jul 2;15:22600. doi: 10.1038/s41598-025-06706-2 (PMC12217066; doi:10.1038/s41598-025-06706-2)
Supplement: Supplementary file 9 — Supplementary Material 9 [file 41598_2025_6706_MOESM9_ESM.docx]

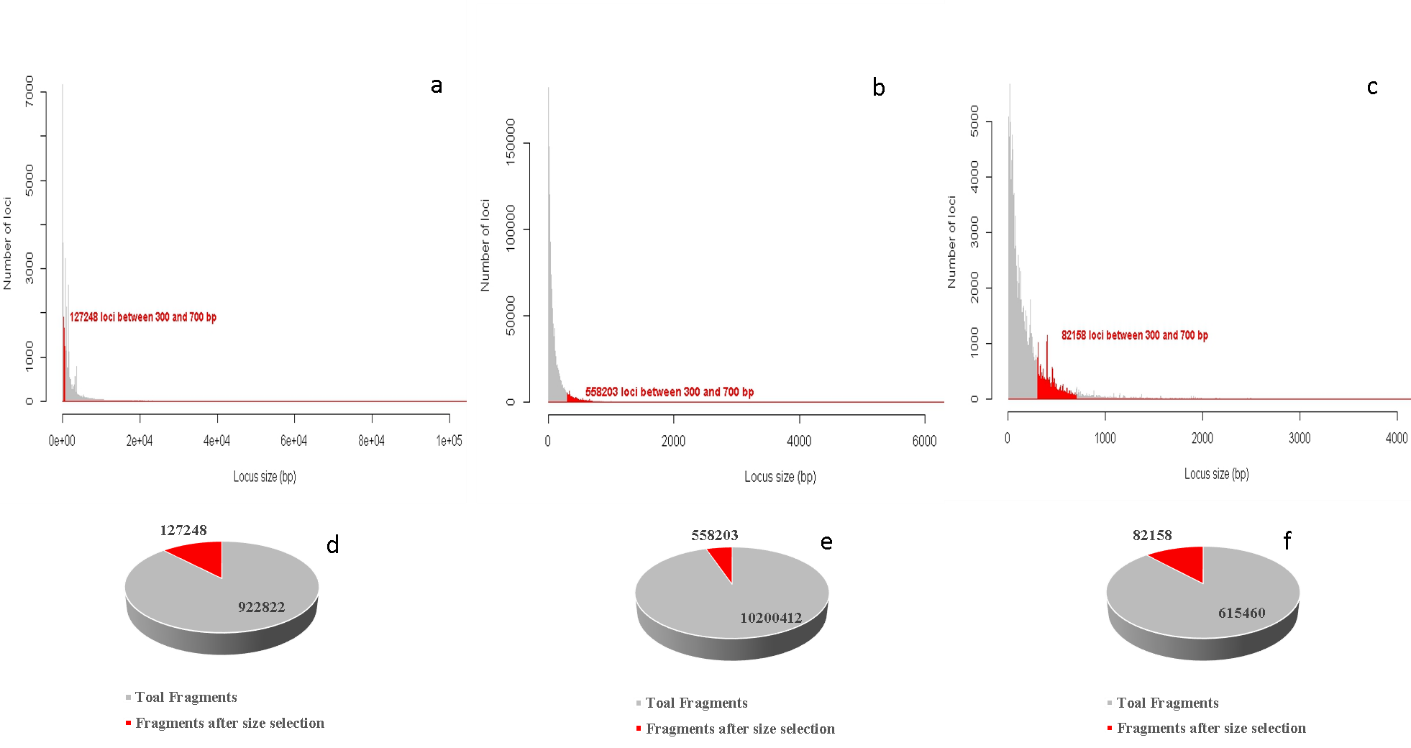


**Fig. S2** Digestion patterns of the safflower genome using ApeKI and two other enzyme combinations, highlighting the distribution of fragments within the 300–700 bp range following enzymatic cleavage. a) ApeKI b) NlaIII_Msel c) EcoRI_Msel. Panels d to f display the fragments remaining after size selection of 300–700 bp, based on the total number of fragments produced: (d) ApeKI, (e) NlaIII_MseI, and (f) EcoRI_MseI.
