# Supplementary material for: Comparative analysis of RAD-seq methods for SNP discovery and genetic diversity assessment in oil seed crop safflower
Source: Sci Rep. 2025 Jul 2;15:22600. doi: 10.1038/s41598-025-06706-2 (PMC12217066; doi:10.1038/s41598-025-06706-2)
Supplement: Supplementary file 11 — Supplementary Material 11 [file 41598_2025_6706_MOESM11_ESM.docx]

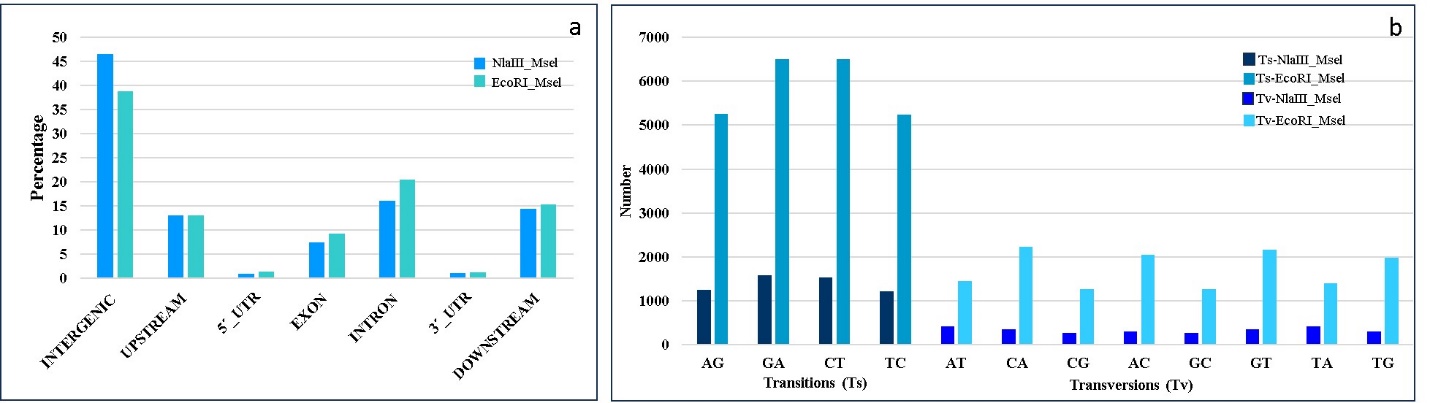


**Fig. S8** a) Distribution of SNPs based on their location in respective predicted gene models in the safflower genome b) Transition (Ts) and Transversion (Tv) counts in NlaIII_Msel and EcoRI_Msel
