# Supplementary material for: Comparative analysis of RAD-seq methods for SNP discovery and genetic diversity assessment in oil seed crop safflower
Source: Sci Rep. 2025 Jul 2;15:22600. doi: 10.1038/s41598-025-06706-2 (PMC12217066; doi:10.1038/s41598-025-06706-2)
Supplement: Supplementary file 12 — Supplementary Material 12 [file 41598_2025_6706_MOESM12_ESM.docx]

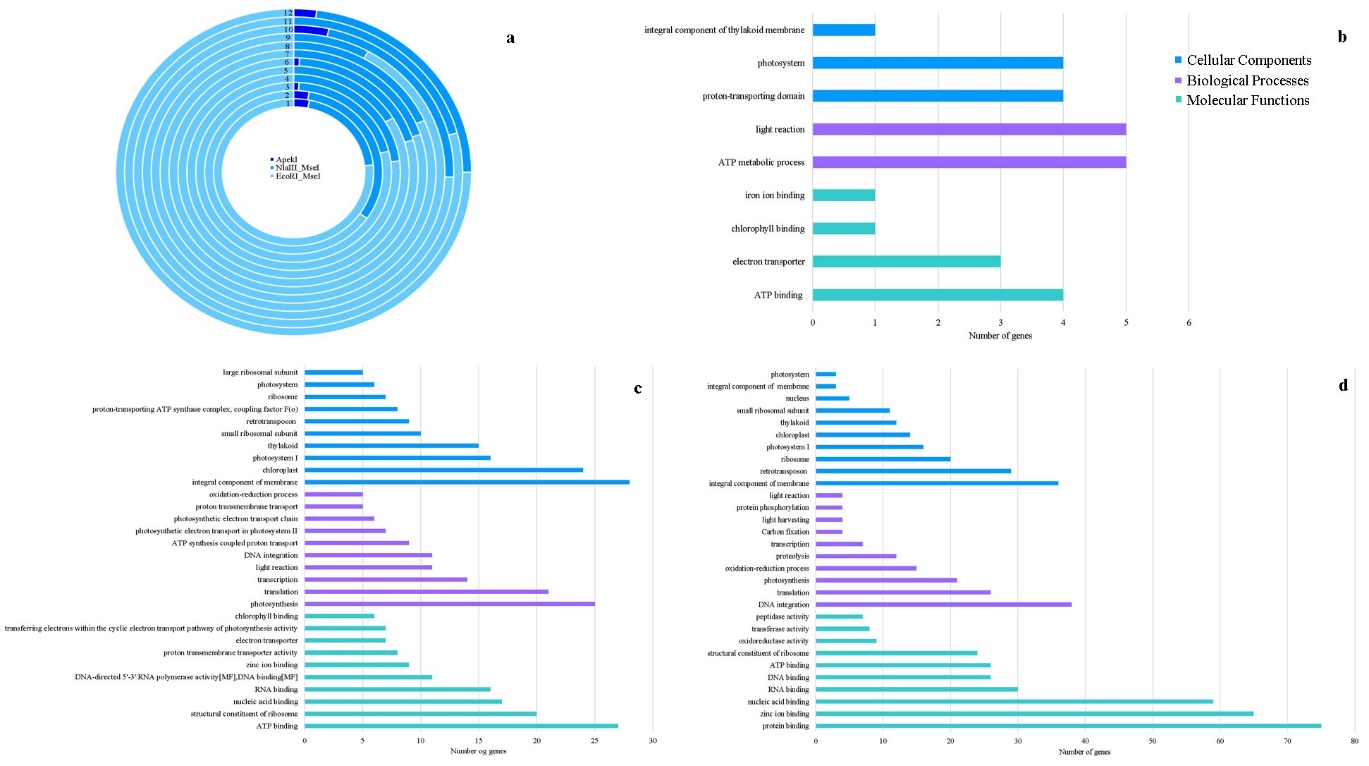


**Fig. S7** a) Chromosome wise coverage of core genes by ApeKI, NlaIII_Msel EcoRI_Msel **Note: The circle's size does not correspond to total number of genes per chromosome.** Gene Ontology b) ApeKI c) NlaIII_Msel d) EcoRI_Msel
